# Inflammation and Its Determinants in Patients With Chronic Kidney Disease: A Study From North Eastern Region of India

**DOI:** 10.7759/cureus.20917

**Published:** 2022-01-04

**Authors:** Anamika Das, Bhupen Barman, Prasanta Bhattacharya, K G Lynrah, Iadarilang Tiewsoh, Pranjal Phukan

**Affiliations:** 1 Internal Medicine, North Eastern Indira Gandhi Regional Institute of Health and Medical Sciences, Shillong, IND; 2 Medicine, North Eastern Indira Gandhi Regional Institute of Health and Medical Sciences, Shillong, IND; 3 Internal Medicine, North Eastern Indira Gandhi Regional Institute of health and Medical Sciences, Shillong, IND; 4 Associate Professor, Department of Radiology, North Eastern Indira Gandhi Regional Institute of Health and Medical Sciences, Shillong, IND

**Keywords:** inflammation, north eastern region, neutrophil: lymphocyte (n:l) ratio, c reactive protein, chronic kidney disease

## Abstract

Background and objective

Chronic kidney disease (CKD) comprises a spectrum of pathophysiologic processes with increased cardiovascular disease (CVD) risk mediated mostly by endothelial dysfunction other than traditional risk factors. The present study is carried out to see the cardiovascular risk in CKD patients with special reference to the determinants of endothelial dysfunction.

Materials and methods

We enrolled 60 CKD patients along with 120 healthy controls in the age group 18-50 years belonging to the same ethnicity and localities. Demographic and clinico-laboratory information including markers of endothelial dysfunction were recorded followed by univariate and multivariate analyses to assess the relationship between CKD and CVD risk.

Results

Cases comprised of 60 CKD patients (mean age = 38.47±8.56 years) including 35 (58%) females and 25 (42%) males. Etiology in 43% of cases was idiopathically followed by diabetes and hypertension (42%) and obstructive uropathy (13%). On comparing the means of inflammatory markers between cases and controls, calcium phosphate product, c-reactive protein (CRP), erythrocyte sedimentation rate (ESR), and neutrophil: lymphocyte (N:L) ratio was found to be significantly higher (p < 0.05) in cases as compared to controls but carotid intima-media thickness (CIMT) and low-density cholesterol (LDL) did not show a significant difference (p < 0.05).

Conclusion

Our study showed uniformly higher levels of inflammatory markers in cases irrespective of age and gender except for LDL and CIMT which uniquely showed a positive correlation with age. CKD patients require appropriate treatment and preventive measures for CVD with a high index of suspicion as endothelial dysfunction cannot be adequately gauged by traditional risk scoring methods.

## Introduction

Chronic kidney disease (CKD) comprises a spectrum of pathophysiologic processes associated with abnormal kidney function and progressive decline in glomerular filtration rate (GFR). There is a rising incidence of chronic kidney disease that is likely to pose major problems for both healthcare and the economy in future years.

Cardiovascular disease (CVD), the leading cause of death, is mostly precipitated by cardiometabolic risk and chronic kidney disease. Traditional risk factors cannot exclusively explain the high prevalence and incidence of cardiovascular disease in chronic kidney disease, therefore other non-traditional risk factors such as endothelial dysfunction, oxidative stress, and insulin resistance have increasingly been studied. The endothelium is the largest organ in the body strategically located between the wall of blood vessels and the bloodstream. Hypertension, inflammation, diabetes-associated factors such as advanced glycated end products, and uremic toxins are some of the prevalent risk factors of endothelial dysfunction in CKD [1-2]. Moreover, endothelial dysfunction is thought to play an important pathophysiological role in cardiovascular complications in CKD.

Due to immense difficulty in assessing the function of the endothelium directly, surrogate markers such as c-reactive protein (CRP), erythrocyte sedimentation rate (ESR), etc are finding place as means to assess endothelial function indirectly albeit with variable specificity and sensitivity [3-4]. Notably, novel easily available biomarkers such as neutrophil: lymphocyte (N:L) ratio are increasingly being validated as prognostic markers for endothelial dysfunction and CVD risk in patients with and without CKD [5]. The carotid intima-media thickness test (CIMT) which measures the thickness of the inner two layers of the carotid artery, i.e. the intima and the media using either high-resolution ultrasound or magnetic resonance imaging (MRI) is one of the newer prognostic non-invasive markers of atherosclerotic cardiovascular disease and shows a linear relationship with future CVD risk [6-8]. Hence, these markers have been included in the study for the assessment of cardiovascular risk in study subjects.

The North Eastern Region (NER) of India has a high incidence and prevalence of chronic kidney disease. However, there is a relative paucity of studies of the incidence, prevalence, and etiological factors contributing to the burden of kidney disease in this region [9-10]. Hence, detailed insight into the disease is imperative at this moment for defining its epidemiology and peculiarities in this region. In this background, this study was planned to evaluate the cardiovascular risk in CKD patients with a special reference to the determinants of endothelial dysfunction.

## Materials and methods

Study design and sample size

A hospital-based analytical cross-sectional study was conducted, involving two groups of subjects, namely (i) a case group (consisting of CKD patients), and (ii) a control group (consisting of healthy individuals) in a tertiary care teaching hospital at Shillong, Meghalaya in the northeastern region of India.

The study was powered at 80% with a two-sided α = 0.05. The prevalence of endothelial dysfunction in the CKD was considered as 50% based upon previous data, and it was conservatively expected to detect an odds ratio (OR) of at least four. Based upon these assumptions, it was found that at least 50 cases and 100 controls would be needed for each group if the cases and controls were to be selected at a 1:2 ratio. These sample size calculations were performed using OpenEpi v3.0.1. Taking into consideration a non-response rate of 20%, the sample size was estimated to be 60 cases and 120 controls.

Study participants and data collection

On the basis of the above sample size estimations, 60 patients with CKD (diagnosed as per CKD-EPI criteria) in the age group of 18-50 years, were recruited prospectively for the case group from January 2017 to December 2017 from the medicine out-patient department (OPD). The exclusion criteria were patients with clinical suspicion or investigations showing acute or chronic infections such as urinary tract infection, lower respiratory tract infection, or tuberculosis, patients who have known chronic inflammatory conditions such as rheumatoid arthritis, systemic lupus erythematosus, etc., patients below 18 years and above 50 years. For comparison, 120 healthy individuals (who were in good health, and without a history of autoimmune disorders, hepatic or renal disorder) from similar socio-economic backgrounds and from the same ethnicity and geographical areas were enrolled in the control group for the study during the same period.

Relevant demographic and clinical details were taken and entered as per a pre-tested proforma. Demographic data included name, age, sex, religion, occupation, and marital status. Detailed and relevant history was obtained along with clinical examination comprising of measurement of weight, height, blood pressure, general physical examination, systemic examination, etc. were carried out. GFR was calculated by the CKD-EPI formula. A case was said to have diabetes based on self-report or if diagnosed with diabetes during biochemical analysis. A case was said to have hypertension based on self-report or detection of blood pressure > 140/90 mmHg on two separate occasions. Blood was collected from the patients under sterile precautions and the following laboratory parameters were tested: complete hemogram including ESR, blood urea and serum creatinine, Liver function test, serum electrolytes, lipid profile, c-reactive protein. Blood and urine cultures were sent for all cases to rule out the presence of subclinical infections.

Carotid intima medial thickness (IMT) was assessed by Doppler ultrasound of bilateral common carotid arteries. It was measured at three different sites on each side and the average of the three readings was taken. The normal carotid IMT is 0.74 ± 0.14 mm. Some literature also said that carotid IMT < 0.8 mm is associated with normal individuals, and a value ≥1 mm is associated with atherosclerosis. The collected data was entered in Microsoft Excel (Microsoft Corporation 2010).

Statistical analysis

The data were analyzed using Statistical Package for the Social Sciences (IBM Corp. Released 2013. IBM SPSS Statistics for Windows, Version 22.0. Armonk, NY: IBM Corp.). Continuous data were presented as mean [with standard deviation (SD)] or median (with range) depending upon whether they were normally distributed or not. Subsequently, their comparisons among groups were performed by an unpaired t-test, the Mann-Whitney U test, one-sided analysis of variance (ANOVA), or the Kruskal-Wallis test, as appropriate. On the other hand, the categorical data were expressed as count (with percentage), and their comparisons were performed by Fisher’s exact test or the chi-square (χ2)-test, as appropriate. In all calculations, a two-tailed p-value < 0.05 was regarded as statistically significant.

Ethics

The study complied with the tenets of the Helsinki Declaration and it was approved by the Institutional Ethics vide letter No. NEIGR/IEC/2017/20 dated 22/05/2017 and informed written consent was taken from all participants in the study. Voluntary informed written consent was procured from all the participants of the study.

## Results

Our study included 60 patients with chronic kidney disease and compared it with 120 controls. Cases had a mean age of 38.47±8.56 years of which 35 (58%) were female and 25 (42%) were male. The majority of the cases of CKD included in the study were in stage 5 (76.67%) with only eight cases (13.33%) in Stage 4 and six cases (10%) in stage 3. In the majority of cases (43%) from the study, group etiology could not be found, whereas diabetes and hypertension were found in 42% of cases followed by obstructive uropathy in 13% of cases.

Our study showed a significant disease burden especially in the younger population as 53% of the cases were below 40 years of age. The patients in the case group were comparable to subjects in the control group (n = 120) with respect to age (p = 0.11) and sex (p = 0.375) composition (Table 1). The cases showed a significantly lower body mass index (BMI) level (20.27±3.38 kg/m2) as compared to controls (21.74±2.88 kg/m2) with a p-value of 0.003. Mean hemoglobin and albumin levels were significantly lower in cases as compared to controls with p < 0.0001 for both which can be explained by the overall poorer nutritional state in CKD.

**Table 1:** Comparison of baseline characteristics of cases and controls BMI: body mass index

		Type		Total	P value
		Controls	Cases		
Age group (Years)	18-30	41	11	52	
	31-40	38	21	59	
	41-50	41	28	69	
Mean Age (Years)		35.61±9.587	38.47±8.568		0.11
Sex	Male	58	25	83	0.375
	Female	62	35	97	
BMI (Kg/m^2^)		21.74±2.88	20.27±3.38		0.003
Mean Hemoglobin(g/dl)		13.49±1.69	8.14±2.3		0.000
Mean Albumin(g/dl)		4.40±0.57	3.08±0.09		0.000

The mean value of study variables is given in Table 2. 

**Table 2:** Means of study variables CRP: c-reactive protein, CAL-PHOS: calcium phosphate, CIMT: carotid intima-media thickness test, LDL: low-density cholesterol, ESR: erythrocyte sedimentation rate, N:L: neutrophil: lymphocyte

Variables	Type	Number	Mean	Std. Deviation	Std. Error Mean
CRP	Control	120	7.93	7.95	.7260
	Cases	60	28.20	22.33	2.88
CAL-PHOS	Control	120	32.70	7.62	.6959
	Cases	60	48.12	17.46	2.25
CIMT	Control	120	.053	.011	.0010
	Cases	60	.052	.010	.0013
LDL	Control	120	101.26	35.88	3.27
	Cases	54	100.27	36.42	4.95
ESR	Control	120	22.13	17.02	1.55
	Cases	60	48.48	24.60	3.17
N:L ratio	Control	120	2.20	1.30	.1180
	Cases	60	7.39	5.80	.7490

The t-test was performed to compare the means of the study variables among cases and controls. On comparing the means of the above-enlisted variables between cases and controls, calcium phosphate product, CRP, ESR, and N:L ratios were found to be significantly higher in cases as compared to controls. However, the same relation didn’t manifest in the comparison of CIMT and low-density cholesterol (LDL) between cases and controls. The p-value for both is > 0.05 suggesting that they are not significantly different from each other. BMI, in turn, was found to be higher in the controls group with a significant difference (Table 3).

**Table 3:** Comparison of means of study variables of cases and controls CRP: c-reactive protein, CAL-PHOS: calcium phosphate, CIMT: carotid intima-media thickness test, LDL: low-density cholesterol, ESR: erythrocyte sedimentation rate, N:L: neutrophil: lymphocyte

INDEPENDENT SAMPLES TEST									
	Levene's Test for Equality of Variances		t-test for Equality of Means						
	F	Sig.	t	df	Sig. (2-tailed)	Mean Difference	Std. Error Difference	95% CI of the Difference	
								Lower	Upper
CRP	98.125	.000	-8.895	178	.000	-20.27	2.27	-24.76	-15.77
CAL-PHOS	51.683	.000	-8.243	178	.000	-15.41	1.87	-19.11	-11.72
CIMT	1.492	.223	.276	178	.783	.0005	.0018	-.0030	.0040
LDL	.272	.602	.168	172	.867	.9897	5.90	-10.67	12.65
ESR	11.516	.001	-8.391	178	.000	-26.35	3.14	-32.54	-20.15
N:L ratio	78.858	.000	-9.359	178	.000	-5.19	.5545	-6.28	-4.09

Following this multivariate analysis was carried out to assess the impact of age and gender on the study variables in addition to the presence or absence of chronic kidney disease. On multivariate analysis, it was found that the cases group had statistically significant higher values of ESR, CRP, N:L ratio, and calcium phosphate product as compared to controls with insignificant differences in terms of LDL and CIMT. Combined analysis with age group and sex did not yield similar significant results implying that the study variables depended mostly on the presence or absence of CKD. This implies that irrespective of age group and gender the aforementioned study variables have statistically significant higher values in the cases group as compared to controls (Table 4).

**Table 4:** Multivariate analysis between cases and controls with relation to age and gender CRP: c-reactive protein, CIMT: carotid intima-media thickness test, LDL: low-density cholesterol, ESR: erythrocyte sedimentation rate, N:L: neutrophil: lymphocyte

MULTIVARIATE ANALYSIS						
Source	Dependent Variable	Type III Sum of Squares	df	Mean Square	F	Sig.
Age Group	CRP	1130.297	2	565.148	3.031	.051
	LDL	86.502	2	43.251	.032	.968
	CIMT(AVERAGE)	.006	2	.003	38.302	.000
	N:L RATIO	15.587	2	7.794	.797	.453
	CALCIUM-PHOS PRODUCT	594.183	2	297.092	2.109	.125
	ESR	3050.507	2	1525.253	4.897	.009
Sex	CRP	1554.717	1	1554.717	8.339	.004
	LDL	817.687	1	817.687	.609	.436
	CIMT(AVERAGE)	.000	1	.000	1.449	.230
	N:L RATIO	49.320	1	49.320	5.041	.026
	CALCIUM-PHOS PRODUCT	262.977	1	262.977	1.867	.174
	ESR	2886.404	1	2886.404	9.267	.003
Type * Age Group	CRP	1022.473	2	511.237	2.742	.067
	LDL	152.824	2	76.412	.057	.945
	CIMT(AVERAGE)	.000	2	.000	2.747	.067
	N:L RATIO	32.706	2	16.353	1.671	.191
	CALCIUM-PHOS PRODUCT	692.023	2	346.012	2.457	.089
	ESR	681.005	2	340.502	1.093	.338
Type * Sex	CRP	1255.661	1	1255.661	6.735	.010
	LDL	186.698	1	186.698	.139	.710
	CIMT(AVERAGE)	4.520	1	4.520	.056	.813
	N:L RATIO	62.464	1	62.464	6.384	.012
	CALCIUM-PHOS PRODUCT	35.741	1	35.741	.254	.615
	ESR	201.568	1	201.568	.647	.422
Type * Age Group * Sex	CRP	402.510	2	201.255	1.079	.342
	LDL	5.770	2	2.885	.002	.998
	CIMT(AVERAGE)	.000	2	6.019	.744	.477
	N:L RATIO	6.248	2	3.124	.319	.727
	CALCIUM-PHOS PRODUCT	65.642	2	32.821	.233	.792
	ESR	1589.993	2	794.997	2.552	.081

## Discussion

Our study had diabetes and hypertension as one of the most common causes of CKDs (41.67%). However, an almost equally large population presented with idiopathic CKD (43.3%), i.e on evaluation, the exact predisposing cause couldn’t be ascertained. This can be attributed to the lack of screening, late diagnosis or referral, and lack of health awareness in the community. Many of the cases had shrunken kidneys with advanced uremia at presentation not amenable for a renal biopsy. Also, the presentation of CKD in relatively younger patients as evidenced by 53% of cases being < 40 years should encourage the health care provider to evaluate in the lines of glomerulonephritis, interstitial nephritis, etc. which are common causes of CKD in the younger population. This is however another challenge in the resource-poor settings in the Northeastern states of India.

Markers of inflammation and its determinants

Among the cases, 75% (45) had a raised CRP level, and the mean CRP among the cases was 28.2+/-22.3 mg/L, which was significantly higher (p < 0.05) than the mean CRP in the control group (7.93+/-7.95 mg/L). The findings were confirmed via t-test as well as multivariate analysis which suggested CRP to be higher in the cases group irrespective of other study variables such as age and gender. Compared to several other studies conducted on the prevalence of CRP in CKD patients, our study showed both a higher prevalence as well as a higher mean CRP level [11-18].

ESR, N:L ratio, calcium phosphate product which was the other surrogate markers of endothelial dysfunction included in our study showed significantly higher values in cases as compared to controls. 80% of the cases had an elevated ESR level. The findings correlated to other studies conducted on levels of ESR in CKD patients which have reported a 75% to 100% prevalence of raised ESR in CKD patients [19-22]. Neutrophil lymphocyte ratio was found to be 7.39+/-5.80 in cases and was significantly higher (p = 0.000) than in controls (2.2+/-1.3). Higher N:L ratio has been proven to be an important predictor of cardiovascular and all-cause mortality in CKD patients thus corroborating our findings of raised inflammatory markers in our case population and implying their increased cardiovascular mortality in turn. Calcium phosphate product which is also emerging as a predictor of cardiovascular risk in CKD patients was also found to be significantly raised in the cases group as compared to the control group (48.12+/-17.46 v/s 32.70+/-7.62:p=0.000).

On the other hand, CIMT showed no significant difference in the case and control groups (p=0.783). However, on further analysis, CIMT showed a positive correlation with age irrespective of cases or controls. Though CIMT is a marker of cardiovascular risk it didn’t show the same relation as other markers. This can be explained by the lower mean age in our study population as CIMT is strongly dependent on age apart from other factors. LDL levels similarly showed no significant difference among our cases and controls (p=0.867). This can again be explained by the fact that dyslipidemia in CKD is uniquely represented by increased triglycerides and lower high-density lipoprotein (HDL) unlike in the general population where LDL levels are independent markers of cardiovascular morbidity [6-8,23]. This is another indicator of the inability of common traditional cardiovascular mortality measures to assess the same in CKD patients.

Limitations of the study

The study was conducted on a relatively small study population due to constraints of time. The presence of subclinical infections resulting in higher levels of inflammatory markers couldn’t be completely ruled out though every effort was taken to exclude cases with the slightest hint of the presence of any local or systemic infections at the time of the study.

## Conclusions

Chronic kidney disease is always a challenge to the treating physician. With the ever-increasing burden and younger population being diagnosed with the disease, the morbidity owing to it is increasing. Cardiovascular disease and risk assessment though being an established field are still evolving especially in the context of chronic inflammation and endothelial dysfunction contributing to the same. The search for the ideal biomarker to assess endothelial dysfunction is continuing and still has a long way to go. Until then a high index of suspicion is required in order to treat the cardiovascular complications of CKD as they may not show a correlation with age and traditional risk factors. In fact, in CKD patients, cardiovascular disease is one complication that can never be overdiagnosed. The need for further research is imperative to bring into light the causes of chronic kidney disease plaguing the region which can aid to prevent or delay progression to end-stage kidney disease and treatment of respective causes whenever possible.
